# Synthesis and characterization of rice husk biochar via hydrothermal carbonization for wastewater treatment and biofuel production

**DOI:** 10.1038/s41598-020-75936-3

**Published:** 2020-11-02

**Authors:** Nazia Hossain, Sabzoi Nizamuddin, Gregory Griffin, Periasamy Selvakannan, Nabisab Mujawar Mubarak, Teuku Meurah Indra Mahlia

**Affiliations:** 1 School of Engineering, RMIT University, Melbourne, VIC 3001 Australia; 2 Civil and Infrastructure Engineering, School of Engineering, RMIT University, Melbourne, VIC 3001 Australia; 3 School of Science, RMIT University, Melbourne, VIC 3001 Australia; 4 Department of Chemical Engineering, Faculty of Engineering and Science, Curtin University, 98009 Miri, Sarawak Malaysia; 5 School of Information, Systems and Modelling, Faculty of Engineering and Information Technology, University of Technology Sydney, Sydney, NSW 2007 Australia

**Keywords:** Environmental sciences, Energy science and technology, Materials science

## Abstract

The recent implication of circular economy in Australia spurred the demand for waste material utilization for value-added product generations on a commercial scale. Therefore, this experimental study emphasized on agricultural waste biomass, rice husk (RH) as potential feedstock to produce valuable products. Rice husk biochar (RB) was obtained at temperature: 180 °C, pressure: 70 bar, reaction time: 20 min with water via hydrothermal carbonization (HTC), and the obtained biochar yield was 57.9%. Enhancement of zeta potential value from − 30.1 to − 10.6 mV in RB presented the higher suspension stability, and improvement of surface area and porosity in RB demonstrated the wastewater adsorption capacity. Along with that, an increase of crystallinity in RB, 60.5%, also indicates the enhancement of the catalytic performance of the material significantly more favorable to improve the adsorption efficiency of transitional compounds. In contrast, an increase of the atomic O/C ratio in RB, 0.51 delineated high breakdown of the cellulosic component, which is favorable for biofuel purpose. 13.98% SiO_2_ reduction in RB confirmed ash content minimization and better quality of fuel properties. Therefore, the rice husk biochar through HTC can be considered a suitable material for further application to treat wastewater and generate bioenergy.

## Introduction

In recent years, the concern of circular economy implementation has been highlighted in Australia, especially after China’s decision to ban the import of waste from foreign countries, including Australia, in January 2018. Therefore, Australia emphasized on effective circular economy throughout the country urgently to prioritize the collection of waste resources, recovery, and re-use of it. The circular economy is defined as the alternative conventional linear model to use the waste resources as long as possible, extract the maximum energy and economic value, recover and regenerate products at the end of the service^1^. The relevant renewable waste-based sources in Australia are agricultural residue (e.g., husk, straw, bagasse), forest residue (e.g., timber waste), municipal waste (e.g., sewage sludge), and industrial wastes (e.g., wastes from food industries, oil industries, and others) covered a significant portion of an overall waste generation of the country^2^. Australia is one of the highest quality rice-growing countries worldwide, and most of the rice is harvested in southern Australia through 1500 rice farms^3^. The average annual rice/paddy production in the country is about 600,000–80,000 tons and a maximum of 1.2 million tons on the cultivation area of 65,000–90,000 hectares^3^. After processing and milling of the rice, a large number of RH (around 20% of total amount of paddy) has been produced from rice milling industries. Some amount of these rice residues is used for the animal feedstock/bedding, and the rest are either burnt or abandoned to the soil^3^. Hence, this study focused on utilizing this waste residue and produce value-added products. In most cases, after milling, RH contains 14–15% moisture content that is deemed as a suitable condition for pre-treatment processes (e.g. chemical, thermal and biological) to produce valuable products for further applications such as energy production (biofuel), wastewater treatment, cement supplements, soil amendment, and others^4–7^. Due to the high pre-treatment cost for liquid fuel production from RH, HTC has been selected for this current research study^8^.

HTC, a thermochemical treatment to convert lignocellulosic biomass into char in hot water (180°–280 °C) under pressurized pressure 2–10 MPa with different range of digestion period. The HTC technique approach has dragged research attention recently over other thermochemical conversion techniques to digest biomass samples and synthesize the organic compounds more efficiently due to low-temperature demand and no requirement of pre-treatment for high moisture impregnated biomass^9–13^. HTC has been preferred for this experimental study because of the shorter reaction period, faster char production, higher energy efficiency, and lower processing cost^14^. HTC has tremendous applications for the conversion of waste biomass such as fruit waste biomass (e.g., rotten apple, apple chip pomace, apple juice pomace, grape pomace) and biofuel feedstock, miscanthus yielded 27–45% (wt%) and 46–78% (wt%) char, respectively^15,16^. Other waste biomass, oil palm shell char through HTC, presented 43–62.5% (wt%) yield, and the energy value of char through HTC elevated 9.87 MJ/kg higher than the raw oil palm shell^17,18^.

HTC has been preferred in this study over other most popular biochar conversion method, pyrolysis, due to the particular economic and environmental perspective. Pyrolysis of biomass requires high temperature, and it is an energy-intensive process compared to HTC^16^. According to previous studies, the minimum temperature requirement for slow pyrolysis, medium pyrolysis, fast pyrolysis, gasification, torrefaction, and HTC are 400 °C, 500 °C, 500 °C, 800 °C, 300 °C, 160 °C, respectively, with different ranges of period intervals^19,20^. These studies also demonstrated another advantage of HTC-derived char, which is the highest wt% of solid product (80%) and lowest wt% of liquid (17%) and gaseous product (3%). Since the main objective of this research was to obtain maximum RB and the RB from HTC is far greater than any other conversion process of solid char. Besides, through HTC, organic waste like rice husk containing carbon content can be converted entirely, which made the carbon efficiency of HTC nearly 100%. Therefore, the total carbon content of RH remains at the final product, and a minimal amount of carbon-containing gases (CO_2_, CH_4_, CO, and others) can be formed, which makes this process one of the most environment-friendly conversion methods. Moreover, HTC is an exothermic process; therefore, RH containing mineral substances (e.g., salts of potassium, phosphorus, ammonium, and others) dissolved in the process liquid (mostly water) can easily be isolated. This process water can be used either for soil amendment or re-used for the HTC for further runs^19,20^.

RH is one of the major agricultural wastes in Australia, and potential applications of this biomass utilization are being emphasized currently^2,3^. Few studies of HTC on RH has been performed previously in different areas of the world to determine mainly the fuel properties upgrade in hydrochar after HTC. Suteerawattananonda et al.^21^ investigated thermo-chemical properties and energy values of RB and ash. Another study of HTC on RH was done by Kalderis et al.^22^ presented that RB was produced to determine the fuel content upgrading such as improvement of higher heating value, volatile matter, fixed carbon, and others. The study also determined the surface area but not adequate data for water treatment application^22^. The previous thermo-chemical characterizations of RB through HTC were not comprehensive and sufficient for multiple purposes, such as wastewater adsorption efficiency and energy production. Therefore, a detailed characterization study of RH and RB has been investigated. The main objective of this study was to determine the wastewater adsorption efficiency and potential of bioenergy in RB via HTC. RH and RB were analysed and compared based on the parameters: physicochemical properties, size distribution, zeta potential, surface area, pore diameter, pore volume, chemical and phase, atomic composition and kinetic energy, morphological, elemental (organic and inorganic), structural, crystallinity and thermal profile.

## Results and discussion

### Physicochemical properties

Bulk density and energy density are significant parameters for optimizing transportation and logistics. These parameters estimate biomass and bioenergy storage per unit area, and project the desired techniques for biomass densification, dust removal, as well as storage and transportation, cost reduction^23–25^. Table 1 showed that the bulk density of RH was 83.34 kg/m^3^, while after HTC, bulk density for RB has been improved to 180 kg/m^3^. The bulk density of RB in this study is comparatively higher than some other popular biomass used for biofuel purposes such as baled straw (145.15 kg/m^3^), wood sawdust (108.86 kg/m^3^), softwood chips (163.29 kg/m^3^)^23,26^. Higher heating value (HHV) and lower heating value (LHV) represented the total amount of possible heat production from the samples, including and excluding the latent heat of vapor, respectively^23^. The HHV of RH and RB is 14.53 MJ/kg and 20.27 MJ/kg, respectively, and the LHV for these samples is 13.27 MJ/kg and 19.02 MJ/kg, respectively. The HHV and LHV of RH are more than other waste biomass, such as rice and wheat straw and softwood waste. However, after conversion into char, HHV and LHV values elevated 6 MJ/kg than raw RH, a similar range with hardwood timber^27^. The moisture content of these samples is below 10%, which indicates the excellent quality of fuel value and less heat requirement for moisture removal.

**Table 1:** Physicochemical properties of untreated RH and RB.

Physicochemical properties	Untreated RH	RB
Bulk density (kg/m^3^)	83.34	180
Moisture content (%)	7.5	4.2
Carbon (%)	25.9	47.2
Hydrogen (%)	4.7	4.2
Nitrogen (%)	0.6	0.9
Sulphur (%)	0.3	0.7
H:C	0.18	0.08
C:N	43.16	52.44
Higher heating value (MJ/kg)	14.53	20.27
Lower heating value (MJ/kg)	13.27	19.02
Energy density (GJ/m^3^)	6.05	10.28
Fuel value index (FVI)	807.70	2448.44
Biomass characteristic index (BCI)	38,549.38	48,582.10

It should be noted that the moisture content (MC) value of the RB is near to the MC value of ideal fuel, sub-bituminous coal (3.21%)^28^. Biomass characteristic index (BCI) and fuel value index (FVI) are used for projection of industrial application of any biomass-based on heat production, flammability, biomass density, and moisture impregnation. Due to the elevated bulk density (BD), HHV, and very low MC, FVI, and BCI of RB have been improved significantly, equal to ideal feedstock for biofuel purpose^26^. After the HTC approach, C content increased substantially in biochar, and the H:C ratio decreased to 0.08 from 0.18, which increased the fuel quality and contaminant adsorption from wastewater^23,29^. The presence of a low amount of N and S content is ubiquitous in RH as well as RB, and that the effect on energy or adsorption efficiency is considered as negligible^23^. Table 1 presented the physicochemical properties of untreated RH and RB.

Table 2 showed the mass yield, energy densification ratio, and energy yield of the RB. The mass yield was 57.9% at 180 °C, which was much higher compared to the fruit waste biomass (27–38% at 190 °C) due to the higher lignin content in RH as well as mass yield is the nearby range with popular biofuel feedstock, miscanthus (78% at 190 °C) through HTC^15^. The energy yield of RB through HTC was relatively high, 80.77%, which was similar to other commercial biofuel feedstock such as miscanthus (87%, 76%, and 71% at 190 °C, 225 °C, and 260 °C, respectively) and much higher than fruit waste biomass (32–64%). The energy densification ratio was in the ideal range with other waste biomass (1.08–1.54)^15^.

**Table 2:** Mass yield, energy densification ratio, and energy yield.

Sample item	Properties
Mass yield, Y_m_ (%)	57.9
Energy densification Ratio (ED_ratio_)	1.39
Energy yield, y_e_ (%)	80.77

### Particle size distribution

**Figure 1:**
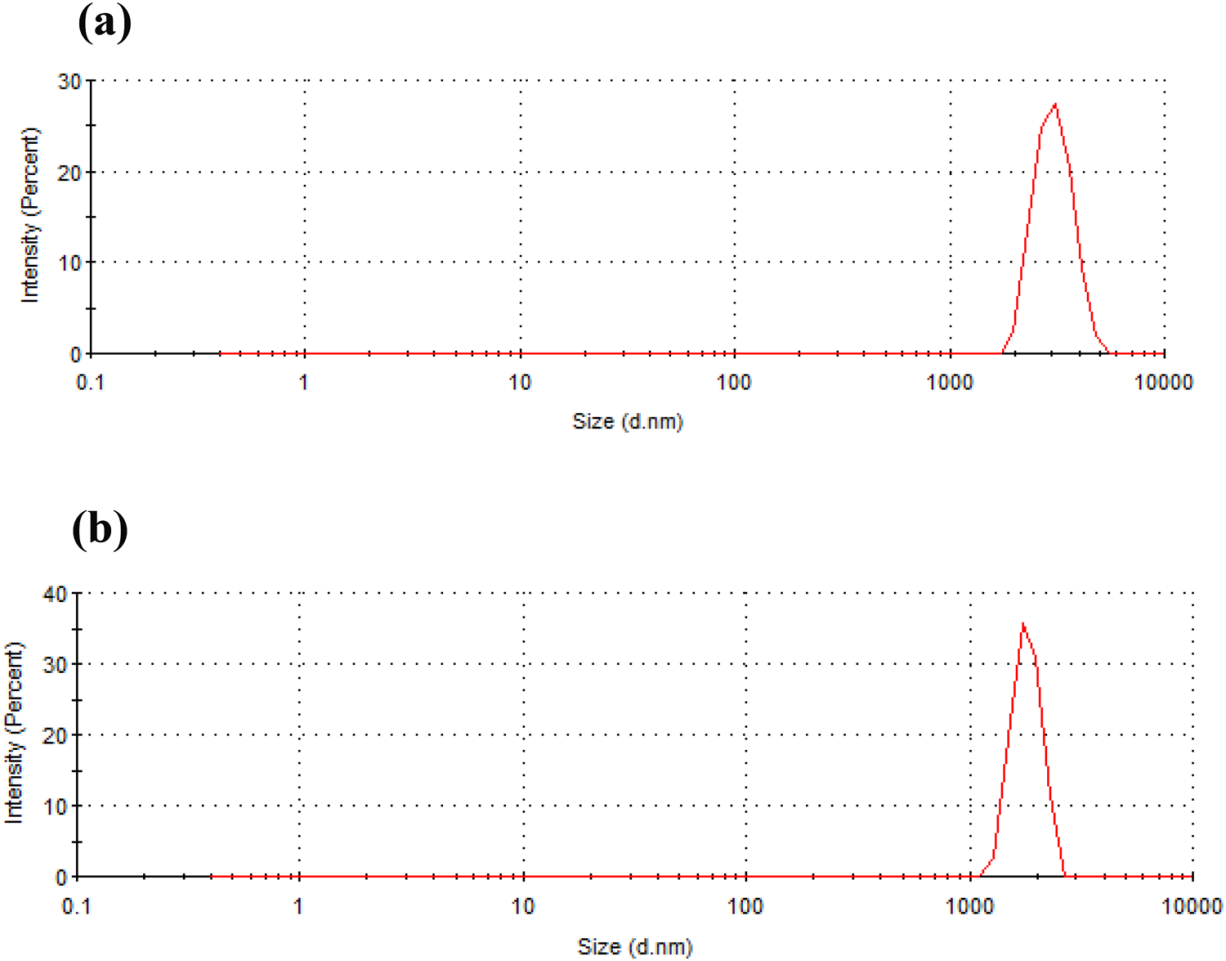
Particle size distribution of RH (**a**) and RB (**b**).

Particle size distribution is a significant analysis to determine the porosity, which indicates the water adsorption and the soil nourishment efficiency. Figure 1a,b presented that the particle size distribution has been decreased for RB (size (d.nm): 2937) in comparison with RH (size (d.nm): 3572). The reduction of the particle size in RB increases the homogeneity of the pore distribution, surface area, total pore volume, and average pore width. These characteristics play a significant role in enhancing the water retention capacity of RB particles. On the other hand, larger particles increase aeration and drainage^30,31^. Previous experimental studies showed that smaller particle size was capable of holding water firmly than larger particles, which very favorable for soil nourishment purposes^30^. With the smaller particle size, the C:N ratio was increased from 43.16 to 52.44 (Table 1), which is related to the alteration of nitrogen and carbon with RB for the soil amendment. C:N ratio is an essential parameter for the nutrient balance and nutrient availability for the survival of microorganisms and plants^30^. Moreover, smaller RB particles can easily interact with the emerging contaminants in wastewater (e.g., industrial, municipal) to form aggregates that trigger contaminant adsorption efficiency from wastewater^30^.

### Zeta potential (ZP) distribution

**Figure 2:**
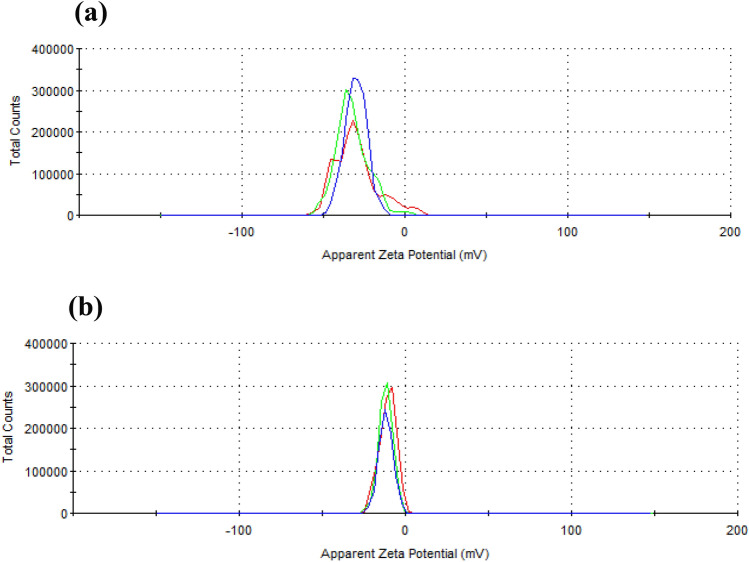
Zeta potential distribution of RH (**a**) and RB (**b**).

ZP is known as surface potential related to surface charge. ZP is a crucial factor to determine the effect of sample particles in suspension such as particle agglomeration, sedimentation, interaction as well as complexation with other media elements^32^. Figure 2a presented the ZP of untreated RH is − 30.1 mV while ZP decreased to − 10.6 mV for RB in Fig. 2b, and the resulting quality for RB has been referred to as ‘good’ by the analyzing software. ‘Good’ via analyzing software indicates the good quality of the zeta potential result due to its’ acceptable zeta deviation of three repeated ZP measurements. According to previous study on ZP analysis, ZP values less negative than − 15 mV (for RB) represents the onset of agglomeration. The threshold region of either coagulation or dispersion exists from approximately − 14 to − 30 mV. ZP values more electronegative than − 30 mV represents sufficient mutual repulsion to result in stability such as no agglomeration^33^.

RH has many polar chemical functional groups that stabilize the particles electrostatically, responsible for its high negative ZP. However, the RB obtained after HTC has less polar functional groups, thereby less ZP. Another notable difference is their size. HTC derived RB particles’ hydrodynamic radius was much smaller than the RH particles’ hydrodynamic radius (Fig. 1b). ZP of the particles implies the extent of the stability of colloids by electrostatic. Simultaneously, colloidal stability can also be achieved by the steric hindrance of bulk chemical groups like polymer colloids. These colloids are stable though they were reported to have less ZP. RB particle size is small, and the carbonized particles stabilize through steric interactions.

The negative ZP in both samples presented the high lignin content^34^. Earlier studies on the effect of ZP on SiO_2_ nanosphere displayed that the higher ZP value represents better stability. The negative charges (cation) on the surface are inevitable for static interaction with other biological and chemical components. The negative charges reflected the electrostatic interactions of sample particles on material surfaces^35^. Therefore, cations presented the enhancement of loading efficiency, adsorption capacity, and stability of the mixture solvent. To note, ZP values above + 30 mV and below − 30 mV are considered as the most potential values that permit the highest stable suspension and state of colloidal systems^35^. Thus, the ZP value (− 10.6 mV) of RB was reported as good through the ZP simulation software.

### Surface area, pore diameter, and pore volume analysis

**Figure 3:**
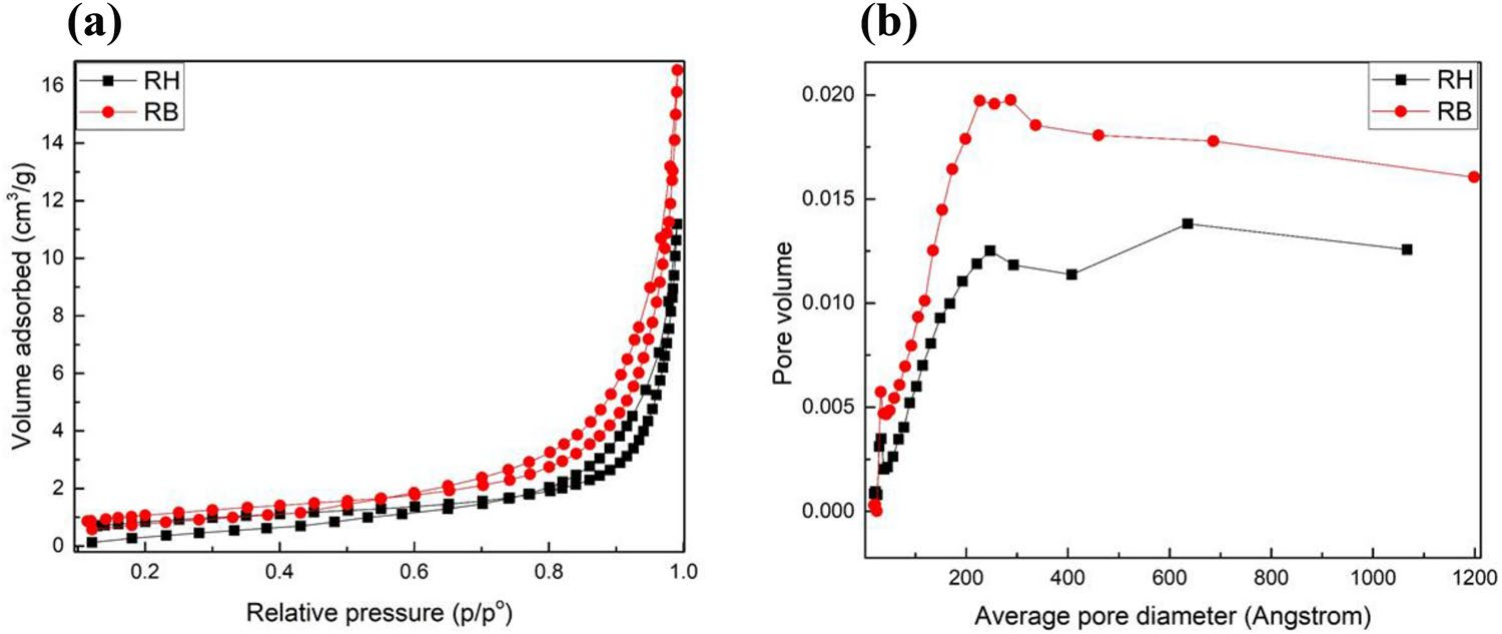
N_2_-sorption isotherms of RH and RB.

Figure 3 showed the sorption isotherms, pore size distribution obtained from the Barrett–Joyner–Halenda (BJH) equation using the desorption branch of the isotherm. The surface area, pore diameter, and pore volume of RH and RB obtained from the isotherms are given in Table 3 BJH method, desorption branch.

**Table 3:** The surface area, pore diameter, and pore volume of RH and RB (BJH method, desorption branch).

Sample name	Surface area m^2^/g	Pore diameter (nm)	Pore volume cm^3^/g
RH	3.5	19.6	0.017
RB	5.02	20.2	0.025

RH and RB exhibited hysteresis in their respective isotherms, which is a clear indication of the presence of mesopores^36,37^. However, surface area, pore diameter, and pore volume of RH were found to increase marginally after HTC, when RH was converted into RB. The increase in surface area, pore diameter, and pore volume after HTC was probably due to the loss of volatile organic compounds, which eventually increase the porosity of the materials. The mesopore filling region (Fig. 3) in the isotherm was observed at lower relative pressure in the case of RB, which is a clear indication of larger pores as compared to the RH. The same trend was observed in the pore size distribution (Fig. 3), wherein the average pore diameter estimated using the BJH equation was found to be higher in the case of RB. From the sorption isotherms, it is clearly understood that HTC of RH was led to the formation of larger pores and increased surface area in the case of RB due to the loss of volatile organic compounds present in the RH^37^.

Though the pore size of RB has been improved after HTC, the improvement is not much significant since the current study applied 180 °C temperature with 20 min without any catalyst addition. Some previous studies presented that with higher temperatures (280°–350 °C) and longer period (10–20 h) conditions, HTC or slow pyrolysis provided higher porous hydro/biochar with significant adsorption capacity^19,20^. However, it would be higher energy-intensive and costly^19^. Therefore, the experimental results of this study recommend higher temperatures and a more extended processing period for HTC in the future to improve the quality of RB besides a good quantity of the final product.

### Morphological analysis

**Figure 4:**
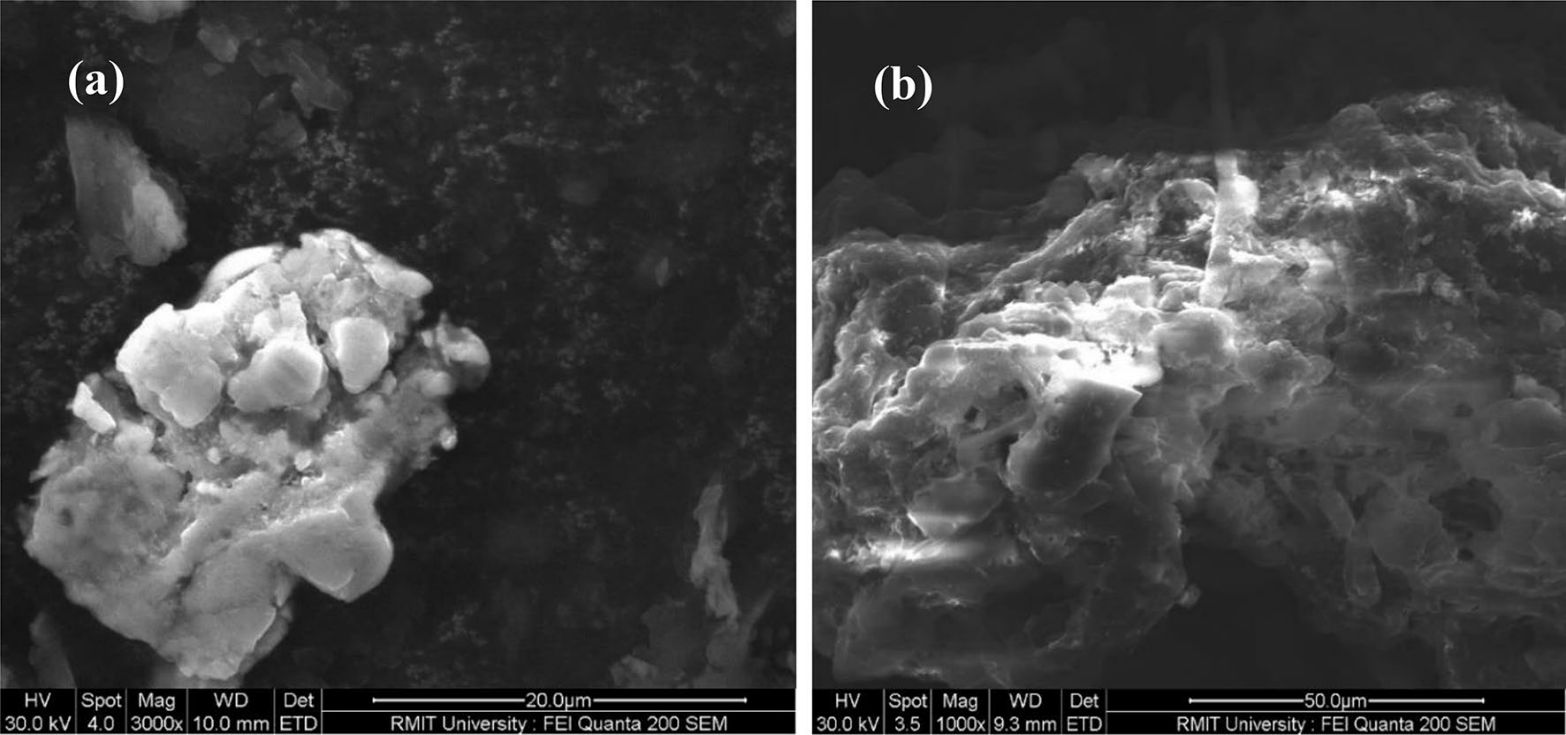
Morphological analysis of RH and RB.

The surface view of RH and RB has been analyzed through scanning electron microscopy (SEM) at 30 kV. Figure 4a presented the RH surface, which looks very congested, non-porous, rigid, and densely formed, while the RB surface in Fig. 4b gave a porous view which is favorable for wastewater. The morphological analyses through SEM supported the enhancement of porosity and surface area based on the Brunauer–Emmett–Teller (BET) analysis and BJH method.

### Chemical and phase analysis

**Figure 5:**
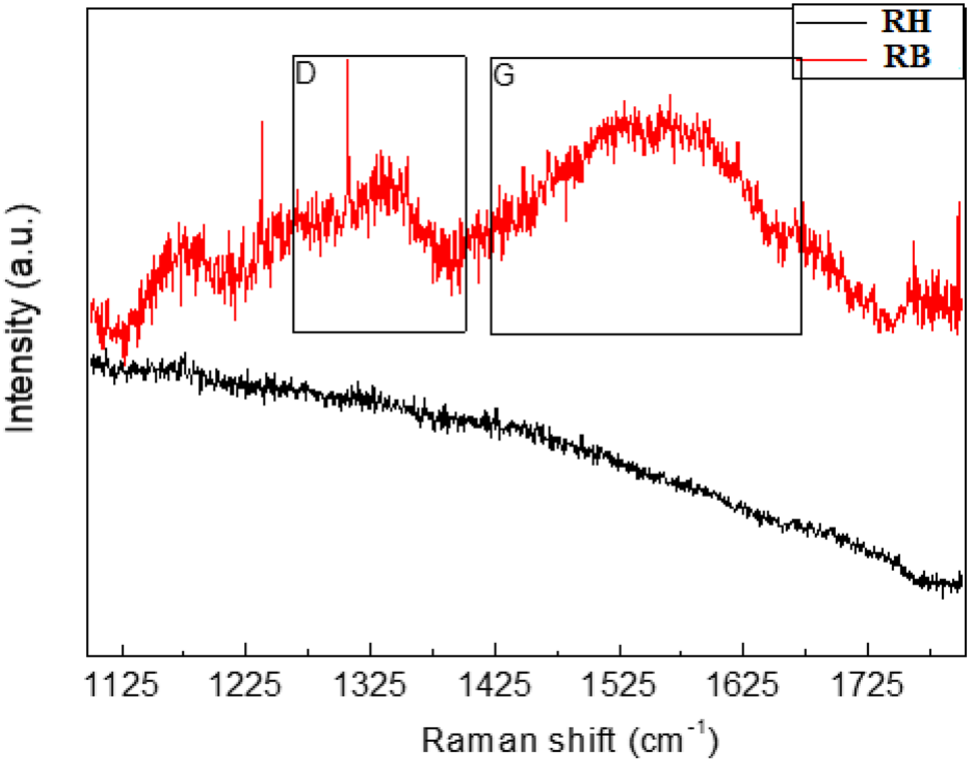
Phase analysis by Raman spectroscopy.

The Raman spectra obtained from the RH and RB showed in Fig. 5 were significantly different. RH didn’t show any intense peaks, which demonstrates the absence of carbon formation. However, RB showed a broad and less intense peak around 1325 cm^−1^, attributed as D-band, from the amorphous carbon (sp^3^ hybridized). The most intense and broadband observed around 1560 cm^−1^, which was assigned to the highly crystalline graphitic carbon (G band). The G band was the characteristic feature of sp^2^ hybridized in-plane carbon–carbon stretching vibration. The frequency and intensity of the ‘D’ and ‘G’ Raman shifts reveal the degree of crystallinity of the nature of carbon and the ratio of ID/IG (at FWHM of D and G bands), which represents the crystalline order of graphite. From the spectra, it was observed that a significantly high proportion of graphite carbon formed during HTC.

### Atomic composition and kinetic energy analysis

**Figure 6:**
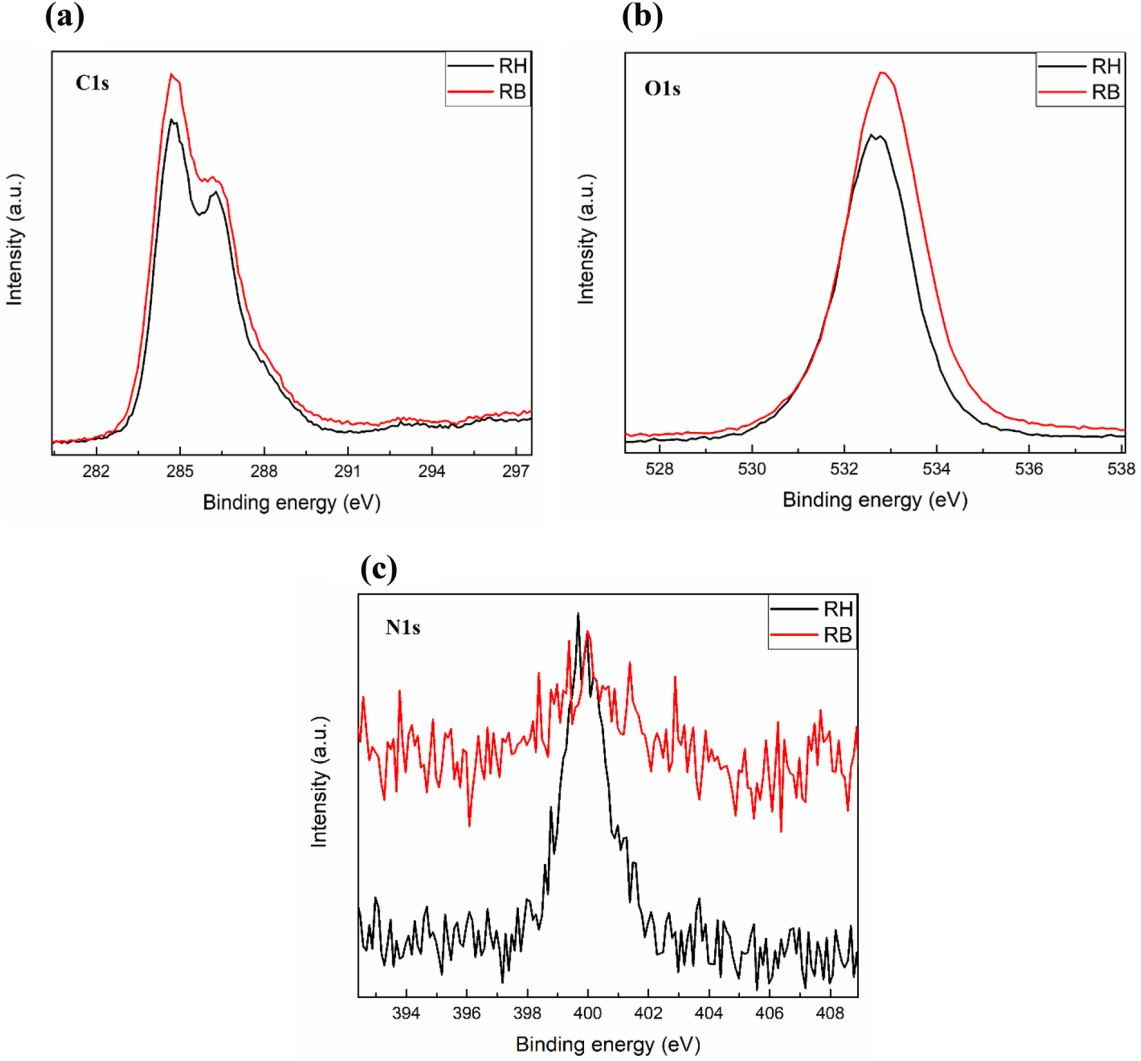
Binding energy analysis of C1s (**a**), O1s (**b**) and N1s (**c**) at RH and RB by XPS.

X-ray photoelectron spectroscopy (XPS) determines the binding energy of electrons in molecules correlated with the distribution valence charges. It provides the data related to the oxidation state of an ion^38^. The XPS spectrum of RH and RB showed in Fig. 6a,b, and Fig. 6c presented the C1s, O1s, and N1s core level spectra, respectively obtained from the RH and RB samples. XPS is a surface-sensitive technique. Therefore, the spectra provide information about the changes in the chemical and oxidation of these three elements after RH was subjected to HTC to produce RB. Significant changes were observed in the N1s core level spectra because of HTC. N1s peak binding energy observed at 400 eV in the case of RH disappeared in the fact of RB. This incident occurred probably due to the loss of nitrogen-containing functional groups present in the RH after HTC. According to Table 1, HTC of RH led to an increase of total nitrogen content from 0.6% in the case of RH to 0.9% in RB. These are the ‘relative nitrogen percentage content’ estimated from both RH and HTC derived RB. HTC process led to the removal of moisture and other volatile compounds from RH. Therefore, the mass of the resultant RB must be lesser than the initial mass of RH. Thus, the estimated relative nitrogen content of the RB must be higher than the relative nitrogen content of the RH. However, the parameter that explains ‘the loss of nitrogen-containing functional groups’ is the C:N ratio (Table 1), which was increased from 43.16 to 52.44, a clear indication of the loss of nitrogen-containing functional groups. This result agreed with the N1s core level spectral results obtained from the RH and RB, wherein the intensity of the N1s signal reduced significantly after HTC.

Similar changes were observed to a lesser extent, in O1s and C1s core level spectra. O1s core level peak binding energy observed at 532 eV, was shifter at 533 eV in RB. This shift is probably due to the change in the chemical state of oxygen-bearing functional groups present in the RH. C1s core level spectra obtained from RH exhibit two significant peaks, which indicate two chemically distinct types of carbon. After HTC, their binding energies didn’t change much, but their relative composition changed. During HTC, there may be a loss of more oxygen-bearing carboxyl groups (–COOH), which was the main reason for the change in the relative composition.

Table 4 presented the atomic composition (%) of O1s, C1s, and O/C ratio. O/C ratio has been upgraded from 0.48 to 0.51 in RB after HTC, which presented the possibility of an intense hydrogen breakdown of crystalline cellulose by HTC. Therefore, free OH- groups were generated, and the O/C ratio increased. An XPS study on lignocellulosic biomass demonstrated that O/C of sugarcane bagasse was 0.27. In contrast, 0.59 O/C ratio was obtained via several pre-treatment methods such as AlCl_3_, FeCl_3_, Al(NO_3_)_3,_ and Fe(NO_3_)_3_ to enhance the enzymatic conversion lignocelluloses for biofuel purpose. For RH, O/C ratio is naturally high while HTC improved more, which is favorable for biofuel purposes^39^.

**Table 4:** Atomic composition (%) analysis of RH and RB.

Samples	O1s	C1s	O/C ratio
RH	32.83	67.16	0.48
RB	34.18	65.81	0.51

### Elemental analysis

According to the X-ray Fluorescence (XRF) analysis in Table 5, the primary constituents of RH were silicon (Si), phosphorus (P), potassium (K), and chlorine (Cl), calcium (Ca), manganese (Mn), and iron (Fe). The minor elements were sodium (Na), magnesium (Mg), aluminum (Al), titanium (Ti), Chromium (Cr), nickel (Ni), copper (Cu), and zinc (Zn). After HTC treatment, Si components reduced, but the amount of nutrient components such as Na, S, Cl, K, Ca, Mn, Mg, Zn, and Fe increased in RB. The elevation of mineral composition in RB of waste biomass (sewage sludge, agricultural biomass waste, wood biomass waste) is responsible for heavy metals (such as Cr^3+^, Cd^2+^, Cu^2+^, Pb^2+^) adsorption from aqueous solution^40^. Previous experimental studies on activated carbon from doum fruit waste improved the adsorption efficiency while RB is used as adsorbents^41^. Besides, the improvement of nutrient elements in RB is an excellent indication for soil amendment and an increase in soil fertility^42^. For the case of oxide compositions in Table 6, SiO_2_ has been reduced from 27.44 to 13.46% after HTC treatment, while nutrient oxides Na_2_O, MgO, K_2_O, MnO, Fe_2_O_3_, ZnO, Al_2_O_3_, SO_3_ increased in amount. The transition metals (e.g., K, Ca, Mn, Fe, Cu, Zn, Cr, and others) and their oxides are usually used to catalyze redox reactions. Therefore, the increase of these metal elements behaves as a catalyst due to the ability to transform oxidation state and consequently, adsorbs other substances more embedded on their surfaces^41^. The presence of significant SiO_2_ in both RH and RB indicates these both materials can be suitable for ceramic products such as structural ceramics, membrane filter for water treatment, insulators, and others. Ceramic membranes can be applied as clarifiers, separators, decontaminants in different industries such as petrochemical, food-based products, and wastewater treatment^42^. A study on waste biomass utilization in India demonstrated that some food-processing sectors (e.g., diaries, beverages) utilized RH for multi-purposes: heat production through boilers, removal from whey protein from whey (separation), and fruit juice clarification^42^. On the other hand, the decrease of Si and SiO_2_ in RB presented low ash content, which is much favorable for heat production by the combustion process through furnace and boiler. Si is deemed as the significant ash constituent, which leads to environmental pollution by fouling and slugging as well as causes corrosion in furnace and boiler during combustion and reduces the life-time of the furnace and evaporator^23^.

**Table 5:** Inorganic elemental composition (concentration) of RH and RB (%) determined by XRF analysis.

Sample	Na	Mg	Al	Si	P	S	Cl	K	Ca	Ti	Cr	Mn	Fe	Ni	Cu	Zn
RH (%)	0.01	0.06	0.01	12.92	0.15	0.08	0.28	1.56	0.4	0.01	0.02	0.21	0.13	0.02	0.01	0.02
RB (%)	0.11	0.1	0.07	6.30	0.15	0.11	1.45	3.26	1.57	0.04	0.01	0.3	0.4	0.01	0.02	0.19

**Table 6:** Oxide composition (concentration) of RH and RB (%) determined by XRF analysis.

Sample	Na_2_O	MgO	SiO_2_	P_2_O_5_	SO_3_	K_2_O	TiO_2_	Cr_2_O_3_	MnO	Fe_2_O_3_	CuO	ZnO	Al_2_O_3_	CaO
RH (%)	0.01	0.11	27.44	0.32	0.17	1.65	0.01	0.03	0.24	0.17	0.01	0.02	0.04	0.47
RB (%)	0.24	0.18	13.46	0.32	0.27	3.73	0.06	0.01	0.36	0.53	0.02	0.22	0.15	-

### Structural analysis

**Figure 7:**
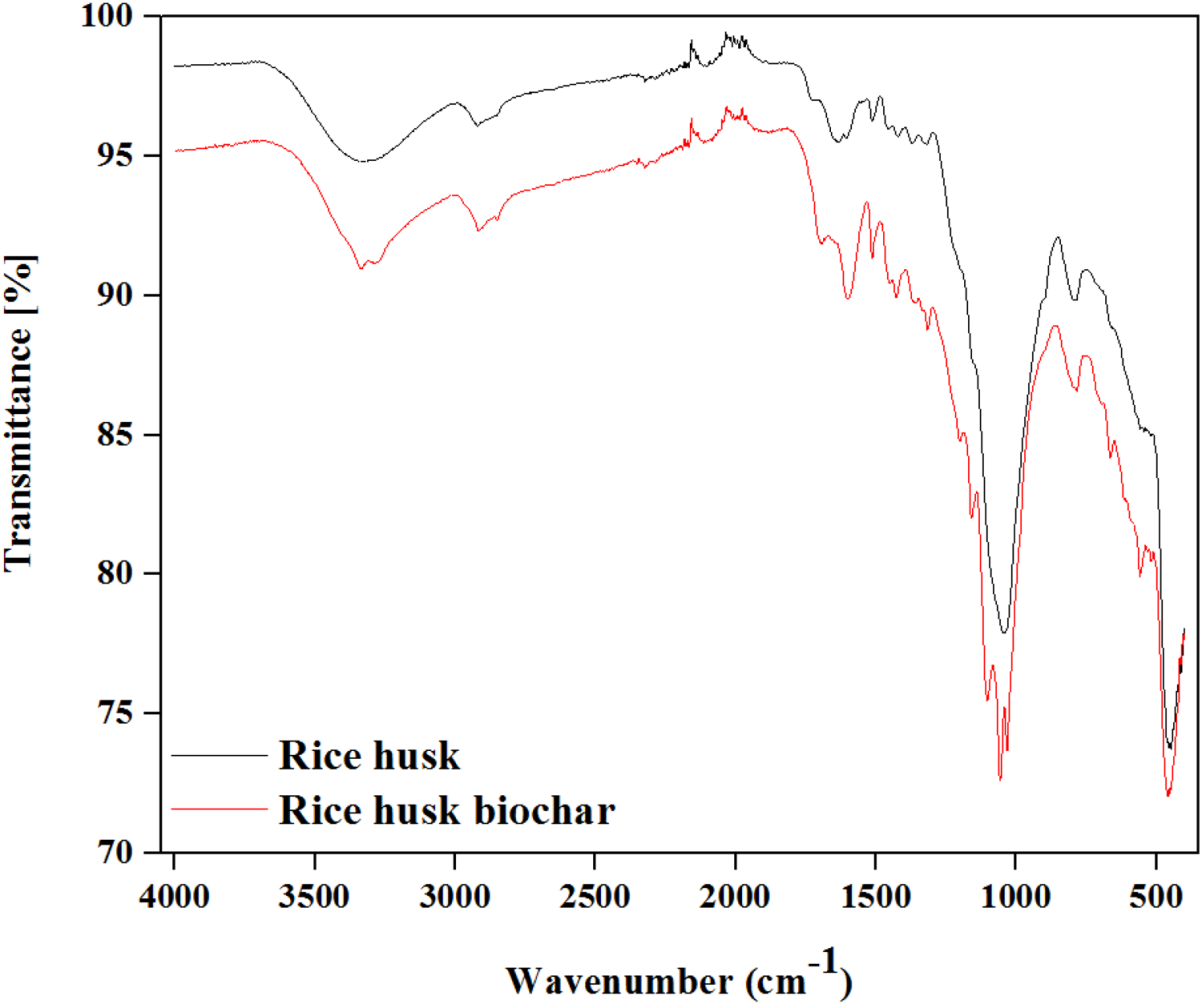
Structural changes by Fourier Transform-Infrared spectroscopy.

Fourier transform infrared (FTIR) spectroscopy is a critical analysis to observe the structural shift changes of chemical compositions. This analysis is the most suitable for identifying the structural similarity of pure material. However, FTIR is also used to analyze changes in biomass after thermo-chemical treatment. Since biomass is heterogeneous material, many peaks can be observed due to bonds of organic compounds. In this study, FTIR analysis was carried out to determine the functional groups of both samples and identify the changes in structural analysis after HTC treatment^40^. The FTIR spectra of both samples were presented in Fig. 7. The peaks observed at 3320–3250 cm^−1^, 1100–1000 cm^−1^, 1065–1015 cm^−1^, and 430–520 cm^−1^ showed O–H stretch, Si–O–Si antisym stretch, C–O stretch, and C–O–C bending, respectively^43^. O–H stretch, C-O stretch, and C–O–C bending have been observed in both RH and RB while Si–O–Si has appeared in RB. Another experimental study of RB from RH and rice straw via pyrolysis presented Si–O–Si antisym stretch since Si is the major component in rice material’s chemical structure. It continued to be present with high temperatures^29^. The most substantial peak for both samples was at C–O stretch and C–O–C bending. A decrease in the intensity of the peaks has appeared in the FTIR spectra after HTC treatment, which happened due to the breakdown of functional groups of lignocellulosic RH after temperature increase. The decrease of H:C ratio (shown in Table 1) manifested this hypothesis. Furthermore, through ZP analysis, the reduction of negative charges on the surface also influences the functional groups –COOH, –COH and –OH to increase the pH in RB and that results from the enhancement of heavy metal adsorption efficiency of RB^40^. Besides, the adsorption efficiency of RB can be elevated more with the incorporation of different catalysts and nano-catalysts in the future to boost the yield and economic viability.

### Crystallinity analysis

**Figure 8:**
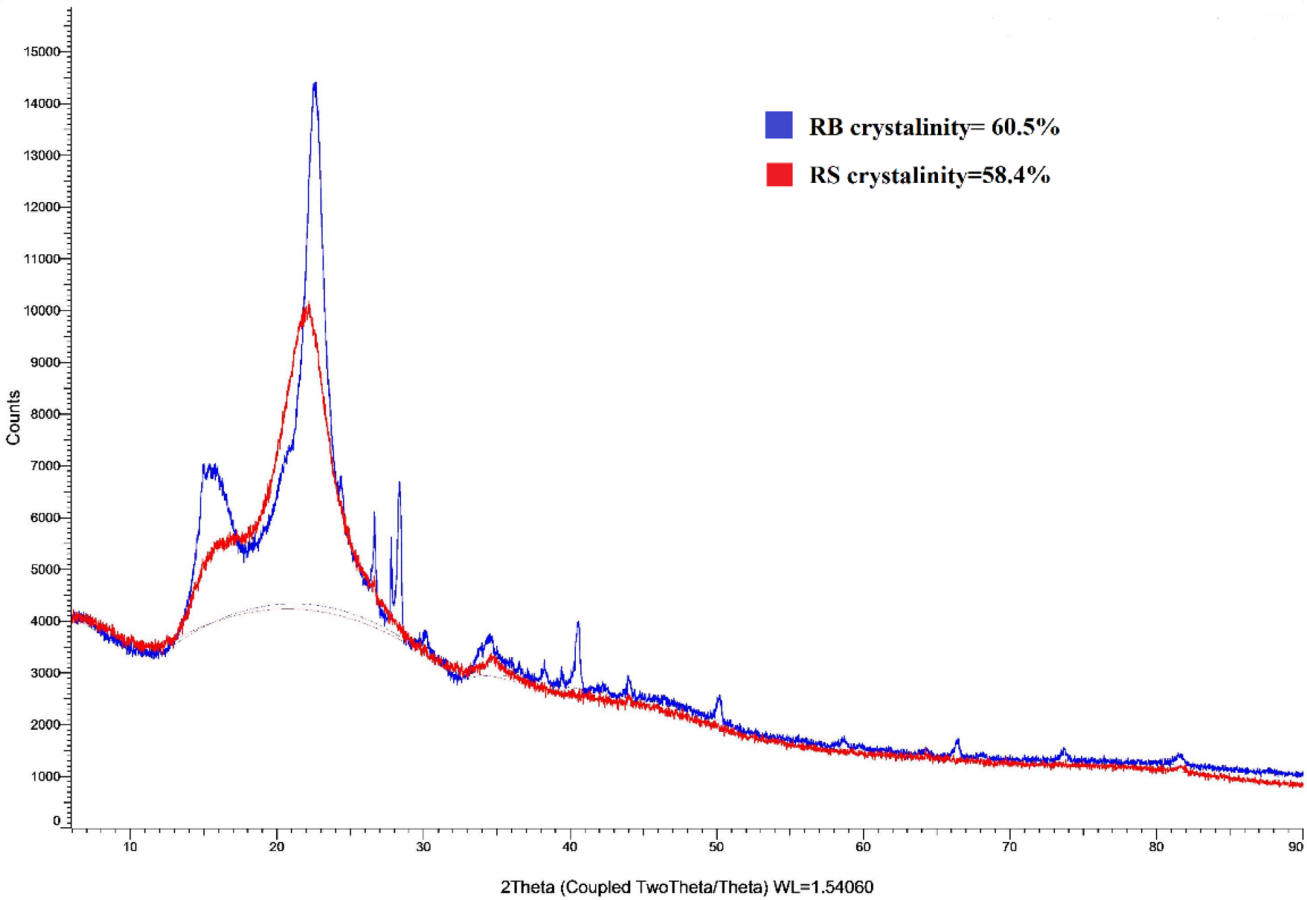
X-ray diffraction pattern of RH and RB.

X-ray diffraction (XRD) analysis presented high crystallinity for both samples, while crystallinity increased in RB after HTC treatment shown in Fig. 8. The crystallinity of RH and RB has been identified as 58.4% and 60.5%, respectively. The crystallinity peak for both samples was obtained between 20° and 25° (at 2θ), indicating significantly less content of amorphous solids, removing organic constituents, and adequate material stability. Based on previous studies, the presence of crystabolite along with quartz (a form of Si) and tridymite in RH boosted the crystallinity after different combustion techniques^42^. High crystallinity contributes to enhancing the catalytic performance of the material significantly^44^. Hence, the higher crystallinity is more favorable for RB/activated carbon to improve the adsorption efficiency of transitional compounds such as Fe^2+^, Cr^3+^, Co^2+^, and others^41^. An experimental study on waste biomass (dried leaves) demonstrated that biomass activated carbon with high crystallinity degraded a common pollutant of industrial wastewater, methylene blue (MB), and the adsorption efficiency was above 98%^44^. Another experimental study on activated tamarind seed presented that the high crystallinity of activated carbon was achieved from potassium (K) compounds, and that effectively adsorbed Fe^3+^, iodine, and MB from wastewater. XRF analysis (shown in Tables 5 and 6) in this study also showcased K was a significant element after Si in both RH and RB, and K and K_2_O accelerated significantly after HTC treatment^45^. Therefore, RB can be projected to be used as ceramic compounds for heavy metals adsorption from wastewater as well as a soil conditioner for the slow-releasing of necessary nutrients.

### Thermogravimetric (TGA) and differential scanning calorimetry (DSC) analysis

**Figure 9:**
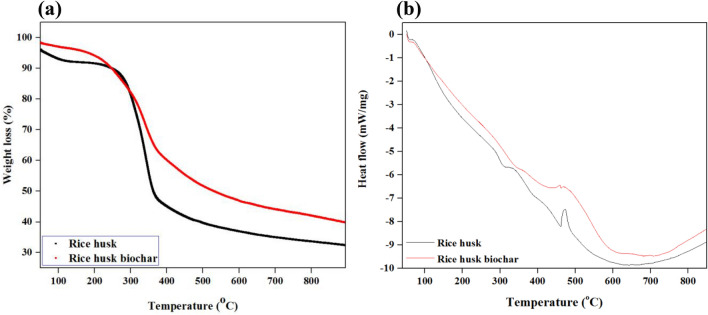
Thermogravimetric analysis (**a**) and differential scanning calorimetry analysis (**b**) of untreated RH and RB.

Figure 9 presented the TGA and DSC analysis of RH and RB, respectively. The parameters: %MC, %volatile matter (VM), %ash content (AC), and %fixed carbon (FC) shown in Table 7 were determined through the TGA output. Sample weight loss vs. temperature change through TG profile in Fig. 9a revealed initial weight loss approximately at 100 °C and 110 °C for RB and RH, respectively, at 20 K/min under inert (N_2_) gas. On the other hand, DSC analysis presented the sample mass loss vs. heat flow of the TGA analysis. This weight loss possibly happened due to the moisture impregnation from the surroundings during the handling and storage process. %MC was determined based on this weight loss^46^. RB contained less MC RH.

**Table 7:** Thermal and oxidative decomposition analysis output by TGA.

Sample	%MC	%VM	%AC	%FC
RH	4.5	56	11.5	28
RB	3	52.5	9.4	35

A slight degradation was observed at 110 °C < T < 280 °C for RH sample while 100 °C < T < 210 °C for RB sample. A scientific consensus mentioned that this slight decomposition phase took place due to the decomposition of low molecular weight products. Therefore, RB contained less low molecular weight products compared to RH^47^. At 280 °C < T < 390 °C, a considerable degradation appeared for both samples caused by thermal decomposition under the inert condition, and that can be considered the peak weight loss range. Volatile matter (VM) was determined based on the thermal decomposition. The volatility of RH was observed roughly 56%, while the char sample has low volatility, 52.5%, because of the carbonization treatment earlier. Immediately after this stage, oxidative decomposition has been initiated at the presence of the air. The sample maintained a slight weight loss to constant weight during the combustion stage.

RH weight loss was almost 8–10% higher compared to RB. AC was identified based on constant weight during combustion. RH and RB both presented a low amount of ash content (11.5% for RH and 9.4% for RB), representing a higher potential for energy profile. Besides, FC values (28% RH and 35% for RB) also revealed adequate-continuous heat generation during the combustion process for fuel applications^24^. Based on the DSC curve in Fig. 9b, within 100–450 °C, heat flow has been concurrently dropping with the sample weight loss. However, heat flow has fluctuated at 450 °C, and this could occur due to the phase change from thermal decomposition to oxidative decomposition. At temperature 640–680 °C, the heat flow was the least for both samples, then heat flow increased slightly and maintained a constant rate. This outcome revealed the least decomposition rate at this temperature. After this stage, heat flow increased slightly but tended to maintain consistent and low heat flow due to the presence of AC^24^. Overall TGA–DSC study represented that RH lost weight and released energy components at a lower temperature than RB. The broader thermal degradation profile of the RH indicated the higher volatility of biomass. Therefore, RH can be projected as a potential feedstock for thermo-chemical conversion processes, while the degradation profile of RB is relatively broader. Less volatility of RB contains the higher potential for adsorption capacity and a cementitious material supplement^48^. Another significant remark of this study is that both experimented samples presented an outstanding thermal profile, which is very close to the thermal profile of leading fossil fuel, bituminous coal (400–420 °C). Hence, it can be summarized that both samples contain the capability to be co-pelletized with coal for industrial implementations to produce heat and energy^28^.

One of the significant challenges to implementing this HTC process in the industrial scale is to fulfil the substantial demand for process water and disposal concerns. Therefore, this study will continue the recirculation of process water in further HTC runs of RH. Based on previous studies, the recirculation of the HTC process water was the most effective technique for heat recovery. It also minimized external heat consumption ten times higher^49^. Since XRF analyses of RB represented the significant presence of various mineral compounds in RB; therefore, the HTC process water is expected to be rich in mineral compounds and total organic carbon compounds. An experimental study of HTC on miscanthus biomass (moisture content < 20%), which has almost similar physiochemical parameters like RH, presented that the HTC process water was highly acidic (pH was in the range of 2.7–3.3) containing various organic acids (mainly acetic acid), total organic carbon and hydroxymethylfurfural particles. These compounds contribute to catalyze the process further, increase process temperature and pressure, and enhance the hydrophobicity of the char. The degraded carbohydrate compounds deposit in the porous structure of the final product and boost energy density. Due to the presence of these compounds, the recirculation of this HTC process water increased the process reaction temperature in the next runs of HTC, mass yields of char improved by 10% in the next two runs, and energy yield amplified up to 80% by subsequent four runs. The HHV of miscanthus HTC-derived char was 26.06 MJ/kg with fresh water as process water while it tuned to 26.64 MJ/kg at the recirculation of the HTC process water at fourth run^50^. The process water re-use can also be implemented for the anaerobic digestion of various biomass to produce other forms of biofuel^51^. Therefore, the continuation of the recirculation of the HTC process water in the industrial plant can notably minimize the use of freshwater and make this approach cost and energy effective and environmentally benign.

## Conclusions

This experimental study addressed waste rice husk utilization for excellent RB yield, 57.9% through the HTC approach. The detailed characterizing parameters presented the sharp elevation of bulk density, energy density, higher and lower heating value, fuel value and biomass characteristic index, lower H/C ratio, outstanding thermal profile improvement through TGA–DSC, which is very favorable for cost-related transportation and logistics and application for biofuel purpose. The reduction of particle size distribution and negative ZP ion (ZP analysis), improvement of surface area and porosity (BET, BJH, and SEM) and O/C atomic composition (XPS analysis), the high proportion of graphite carbon formation in RB (Raman analysis) and enhancement of higher crystallinity (XRD analysis), pH improvement by structural changes (FTIR analysis) in RB demonstrated the increase of adsorption capacity as well as biofuel properties after HTC treatment. The reduction of particle size, improvement of N and S via ultimate analysis, higher C/N ratio, an increase of transitional metal elements through elemental analysis (XRF analysis) described the soil nourishment potential of this biochar. Therefore, the characterizing parameters played significant roles in determining the improvement of energy and adsorption characteristics of the RB so that this char can be used as potential biofuel feedstock and adsorbent in the future.

The choice of using wasted RH from rice mills in Australia to produce RB at low temperatures through HTC presented the opportunity to achieve profit from waste with significant quantities. Since HTC does not demand additional energy and cost for pre-treatment to remove moisture content and is conducted at low temperature and reaction time, this approach can be more realistic and beneficial to be implemented in large-scale applications than other popular biomass conversion techniques such as pyrolysis and gasification. Due to the presence of high cellulosic content and outstanding material stability, this biomass can be integrated with other biofuel feedstock such as timber waste, coal to produce bioenergy. The by-products (bio-oil and aqueous phase) obtained from this HTC is recommended to have experimented further as liquid biofuel as well as bitumen supplement for road-construction and pavement for anti-aging purposes. A comprehensive techno-economic analysis from biomass collection to product implementation, energy balance analysis, and life cycle assessment in further may determine whether this project is commercially viable or not.

## Experimental

### Materials

In this study, RH and RB were characterized. Raw RH was supplied by the Australian private company, Downes Rice Hulls Pty. Ltd. (ABN 62 072 876 629), New South Wales 2710, Australia.

### RH pre-treatment and RB production

The collected RH was brought to the laboratory and washed twice with tapped water, then distilled water to remove the dust and dirt. The clean husk was sieved to remove water and left in the oven with 105 °C for 24 h to remove the moisture content. The dried RH was crushed into 0.5 to 1 mm particle size through MF 10basic microfine grinder drive (IKA Labortechnik, Malaysia) with a speed range of 3.00 to 6.50 rpm and frequency 50/60 Hz. 10 g of RH was experimented via HTC (wet treatment) method at 180 °C and 70 bar with reaction time 20 min by the presence of 300 ml water to produce RB through Parr 5500-4848 series compact reactor (Parr Instrument Company, United States) in an inert N_2_ atmosphere. The operating conditions of RB production via HTC in this study were chosen based on the optimization condition of hydrochar production from RH from the previous study^52^. After 20 min of digestion, the reactor was quenched with the cold tap water, and the temperature was dropped after a while. While the temperature was below 30 °C, the pressure release valve was loosened up, and the gaseous products were fumed out into the fume hood. The remaining mixture of liquid and solid (RB) phases was vacuum filtered through filter paper, Whatman No. 42, to remove the fine char from the liquid phase. The collected RB was washed with distilled water, dried in the oven at 105 °C for 24 h, and the weight of RB has been measured.

### Determination of MC, ultimate analysis (carbon–hydrogen–nitrogen–sulphur), HHV, LHV, BD, ED, FVI, BCI, Y_m_, ED_ratio_ and Y_E_

The MC of both RH and RB was determined, followed by the standard method ASTM D 2974-87. 2 g of fine powder was weighed by analytical balance, then oven-dried at 100 ± 5 °C till reaching constant weight. After having a constant weight of samples, MC was calculated through Eq. (1)^26^.

1 $$ \% MC = \frac{W_{Initial} - W_{Constant}}{W_{Initial}} \times 100\% $$

where W_Initial_ = Initial weight of the sample (g) and W_Constant_ = constant weight of the sample after oven-drying (g).

Ultimate analysis containing Carbon-Hydrogen–Nitrogen-Sulphur (CHNS) content was determined through 2400 series II CHNS elemental analyzer (Perkin Elmer Inc., New Zealand). HHV, LCV, BD, ED, FVI, BCI, Y_m_, ED_ratio_ and Y_E_ were determined through Eqs. (2)–(10), respectively^15,24,26,28,53,54^.

2 $$ HHV = 0.303\left( C \right) + 1.423\left( H \right) $$

3 $$ LCV = HHV - 2.535\left( {9H + MC} \right) $$

4 $$ BD = \frac{m}{V} $$

5 $$ ED = HHV \times BD $$

6 $$ FVI = \frac{HHV \times BD}{{MC\% }} $$

7 $$ BCI = BD \times \left( {100 - MC\% } \right) $$

8 $$ Y_{m} = \frac{{M_{dry biochar} }}{{M_{rice husk} }} \times 100 $$

9 $$ ED_{ratio} = \frac{{HHV_{biochar} }}{{HHV_{rice husk} }} $$

10 $$ Y_{E} = Y_{m} \times ED_{ratio} $$

where HHV = Higher Heating Value (MJ/kg), LHV = Lower Heating Value (MJ/kg), m = Mass of Sample (kg), V = Volume of Sample (m^3^), BD = Bulk Density (kg/m^3^), ED = Energy Density (MJ/m^3^), FVI = Fuel Value Index (no unit), BCI = Biomass characteristic Index (no unit), Y_m_ = Mass Yield (no unit), ED_ratio_ = Energy Densification Ratio (no unit), Y_E_ = Energy Yield (no unit).

### Analysis of particle size and surface charge by ZP

Particle size and surface charge of RH and RB were synthesized through Malvern Zeta Sizer Nano Series (Nano-ZS) (ATA Scientific Pty Ltd., New South Wales, Australia) to determine the sample particle sizing and stability in suspension, respectively. For particle size analysis, the mixture of 0.01 g sample and 1 ml distilled water was transferred into the disposable sizing cuvette (plastic) and placed into the vessel. For surface charge measurement, a mixture of 0.01 g each sample and 0.75 ml distilled water was transferred into clear disposable zeta cell (plastic) and placed into the vessel. Both samples were experimented several times. With Malvern software connected with the Zeta-sizer, Z-average sizing (nm) and zeta potential (mV) were determined.

### Surface area, pore diameter and pore volume analysis by BET and BJH method

BET analysis has been done to determine the surface area, and the BJH method was conducted to identify pore diameter and pore volume. Nitrogen adsorption/desorption isotherms, surface area, and pore volume were acquired at 77.25 K on a Micromeritics ASAP 2010 instrument. HTC derived RB and RH materials were degassed at 125 °C under vacuum for 18 h before collecting sorption isotherms.

### Morphological analysis by (SEM)

Morphological analysis of RH and RB was performed by FEI Quanta 200 Scanning Electron Microscope (Thermo Fisher Scientific, United States) to determine the surface changes in RB. Samples were carbon-coated, then attached to the SEM sample holder and placed carefully on the analyzing chamber, the chamber was closed with a high vacuum environment, and the pressure was auto-controlled by the machine. The sample morphology was analyzed by the computer connected with the SEM machine.

### Chemical and phase analysis by Raman spectroscopy

The chemical structure and phase analysis of both samples were conducted by LabRAM Raman spectrometer (Horiba Instruments Inc., United States) HR evolution equipped with 785 nm (100mW) laser diode and backscattering configuration. The gratings were 600 g/mm with CCD detector (cooled at − 60 °C). The spectral resolution was 10 cm^−1^, 25% laser power, and total acquisition of 5. The Raman spectra in the range of 1100–1800 cm^−1^ were curve-fitted using Raman software.

### Atomic composition and kinetic energy analysis by XPS

Atomic composition percentage and kinetic energy of RH and RB were determined by Thermo k-Alpha X-ray Photoelectron Spectrometer (Thermo Fisher Scientific, United States). The samples were highly vacuumed and placed into the XPS analyzer for 8 h to analyze the samples. The XPS analysis conditions were: X-ray energy- 1486.7 eV (Al k-Alpha), flood gun on pass energy- 50 eV for Hi-res scans, 150 eV for survey, step size- 0.1 eV for hi-res scans and vacuum- 1.2 e^−7^ mBar.

### Analysis of inorganic elemental and oxides composition by XRF

The inorganic elements and elemental oxides compositions were identified through Bruker X-Ray Fluorescence Spectrometer S4 Pioneer (Bruker XRF instruments, Australia). Approximately 4.0 g of the sample was used for this analysis. The elemental analysis was conducted with the presence of Helium (He) gas, and the data were analyzed by the software, Spectra Plus (AXS-34).

### Identification of structural changes by FTIR

Structural changes of RH and RB have been elucidated by the Fourier Transform-Infrared Spectroscopy (FTIR Spectrometer Frontier Instrument, Perkin Elmer Inc., New Zealand). GladiATR single reflection (accessory) with universal ATR diamond crystal has been used. Spectrum (version 10.4.2) software connected to the machine was used to analyze the structural change for both samples.

### Determination of crystallinity by XRD

The crystallinity of RH and RB has been determined by the Powder X-Ray Diffractometer (Bruker D4 Endeavour, United States). 2 g of fine sample powder of each sample was analyzed this experiment. The data were analyzed by the software, Deffrac Eva V4.2, connected to the machine. The crystallinity data have been obtained from the software.

### TGA–DSC analysis

TGA–DSC analysis of RH and RB was performed by NETZSCH 449 High-Temperature DSC-TGA (NETZSCH Australia, Australia). Approximately 7 mg of the fine sample was analyzed for this experiment. The experiment was carried out at 20 K/min under inert (N_2_) gas and oxidative gas (O_2_) gas flowrate, 50 ml/min, and 20 ml/min, respectively, at the suitable condition for the experiment. The gas environment was pre-set as 50kp for N_2_ and 1 bar for O_2_. N_2_ and O_2_ were applied for thermal decomposition and oxidative decomposition, respectively. The temperature, weight loss, and differential scanning calorimetry details were recorded by TGA–DSC software NETZSCH 449. From the analyzed data, the sample mass loss was determined in every stage of combustion, and the percentages of MC, VM, and AC were calculated. The Eq. (11) calculated FC^54^.

11 $$ \% FC = 100 - \left( {\% MC + \% VM + \% AC} \right) $$
